# Testing the efficacy of brief alcohol interventions provided through different delivery channels: study design

**DOI:** 10.1186/1940-0640-8-S1-A70

**Published:** 2013-09-04

**Authors:** Inga Schnuerer, Sophie Baumann, Katja Haberecht, Elke Bandelin, Kornelia Bruss, Stefanie Tobschall, Ulrich John, Beate Gaertner, Jennis Freyer-Adam

**Affiliations:** 1 Institute of Epidemiology and Social Medicine, University Medicine Greifswald, Greifswald, Germany; 2 Institute of Biometry and Clinical Epidemiology, Charité University Medicine Berlin, Berlin, Germany; 3 Department of Epidemiology and Health Monitoring, Robert Koch Institute, Berlin, Germany

## 

In the past 20 years, research has shown that brief interventions are effective in reducing health risk behaviors. Now we are in the second generation of brief intervention trials, investigating how to improve efficacy, including the investigation of cost-saving communication channels. Our aim was to investigate whether motivationally tailored interventions are more effective in reducing unhealthy alcohol use when delivered by computer or in person. Proactively recruited general hospital inpatients with unhealthy alcohol use (n=975) participated in a randomized controlled trial, and were allocated to one of three groups: 1) individualized theory-based computer generated feedback letters, 2) motivational interviewing based counselling, and 3) assessment only (controls). Both intervention groups received up to three interventions. All groups are followed up at 6, 12, 18 and 24 months. Between February 2011 and December 2012, recruitment and interventions have been completed. We have assessed 10,591 inpatients aged 18-64 years for eligibility. We have screened 6,236 inpatients regarding unhealthy alcohol use, corresponding to 91.1% of those eligible. Of all inpatients with unhealthy alcohol use, 80.6% gave informed consent to participate in the trial. So far, 71.3% to 81.5% of these have participated in the follow-ups. A large study has been successfully implemented at a general hospital. Satisfactory participation rates provide a solid basis to investigate the comparative efficacy of brief alcohol interventions delivered by computer versus in person. The follow-up period of up to 24 months provides an excellent opportunity to investigate gradually increasing effects.

